# How much can we learn from each other? Polish and Hungarian good practices in financing ophthalmology care as a proposal for implementation in Ukraine

**DOI:** 10.1371/journal.pone.0306562

**Published:** 2024-07-09

**Authors:** Barbara Więckowska, Katarzyna Byszek, Marek Rękas, Tetiana Yurochko, Maryna Shevchenko, Olena Skrypnikova, Csaba Dozsa, Melanie Toth

**Affiliations:** 1 Healthcare Innovation Unit, Warsaw School of Economics, Warsaw, Poland; 2 Wojskowy Instytut Medyczny, Państwowy Instytut Badawczy, Warsaw, Poland; 3 School of Public Health, National University of Kyiv-Mohyla Academy, Kiev, Ukraine; 4 Med-Econ Ltd, Budapest, Hungary; Federal Medical Centre, Asaba, NIGERIA

## Abstract

**Objectives:**

The article aims to compare payment schemes for cataract, glaucoma, vitrectomy, cornea transplantations, DME, and AMD across Hungary, Poland, and Ukraine, and to identify implementable practices in Ukraine within the context of ongoing healthcare reforms.

**Methods:**

Researchers used mixed-method research–with legal documents and data analysis on utilisation of ophthalmology services between 2010 and 2019 and in-depth semi structured interviews with fifteen health experts from Hungary, Poland, and Ukraine. Interviewees, five from each country, were representatives from healthcare providers and payers with at least 10 years’ experience in ophthalmology care and knowledge about financing schemes in each country of residence.

**Results:**

We identified significant differences in healthcare delivery and financing of ophthalmology services between Hungary and Poland, despite both countries rely on Diagnosis-Related Group (DRG) based systems for hospital care. Good practices for financing specific eye treatments like cataract, glaucoma, age-related macular degeneration (AMD), diabetic macular edema (DME), cornea transplantations, and vitrectomy are identified. The financing scheme, including financial products and incentives, can influence the volume of treatments. Access to ophthalmic care is a key concern, with differences in treatment schemes between Hungary (ambulatory care) and Poland (hospital care), leading to higher costs and the need for centralization of complex procedures like cornea transplantations.

**Conclusions:**

The article highlights the importance of incentivizing quality improvements and removing financial barriers in Poland, while Hungary should focus on continuous monitoring of treatment methods and flexibility in reimbursement. For Ukraine, the research findings are significant due to ongoing healthcare reform, and the country seeks optimal practices while considering the experiences of other countries.

## 1. Introduction

Eye conditions include a wide and varied array of disorders that impact various parts of the visual system and visual function. Due to their diversity, categorizing these conditions can be difficult; however, one approach is to differentiate between conditions that usually do not cause vision loss and those that potentially lead to visual impairment. Ageing is the primary risk factor for many eye conditions [1]. According to WHO, cataract (33%) and uncorrected refractive errors (42%) are leading causes of avoidable visual impairment. Unoperated cataract and glaucoma are the leading causes of avoidable blindness [1]. In addition to elevated intraocular pressure, which is the only modifiable risk factor, risk factors for glaucoma include heredity, race, age, vascular factors, and myopia [2]. Age-related macular degeneration (AMD) is now acknowledged as a multifaceted genetic disorder where one or multiple genes play a role in determining an individual’s likelihood of developing the condition [3]. There are approximately twenty-one million with diabetic macular edema (DME) worldwide and its incidence increases with the duration of diabetes [4]. Vitrectomy is a surgical procedure used to treat retinal diseases such as trauma, diabetic retinopathy, retinal detachment, macular hole, epiretinal membranes, and vitreoretinal traction syndromes [5]. In the case of corneal diseases, corneal transplants are used [1].

Health needs in ophthalmology pose several challenges to health systems and therefore require searching for the optimal models for financing and organizing healthcare services. The Visegrad countries and Ukraine currently experience problems in the ophthalmology area such as suboptimal number of services and limited access to services [6, 7]. In 2019, according to the State Statistics Service of Ukraine, 24.4% of households faced challenges in accessing medical care or acquiring medicines and medical supplies [6].

The international team of researchers launched research with the support from the Visegrad Fund (“Access to healthcare services in the context of financing mechanisms. The case of ophthalmology,” grant no. 22120107), aimed to understand financing mechanisms that impact the access to healthcare services in ophthalmology in the Visegrad countries and Ukraine. The descriptions of financing mechanisms and comparisons of available data on ophthalmic care between the Czech Republic, Hungary, Poland, Slovakia, and Ukraine were presented in the summary report from the project [7]. This article covers in detail findings that enable to compare financing cataract, glaucoma, vitrectomy, cornea transplantations, DME, and AMD treatments in Hungary, Poland, and Ukraine from the perspective of different stakeholders; and second, to identify practices from Poland and Hungary that were assessed during the qualitative research as helpful for decision makers in Ukraine in the light of ongoing healthcare reforms. The overarching goal of this research is to learn from each other about regulatory and financing solutions to be able to improve, in general, the ophthalmology care and services of these countries.

In this article, the term "good practice" is considered as "a technique or methodology that has proven reliably to lead to a desired result through experience and research" [8] and focuses on the existence of a connection between models of financing ophthalmological services and their impact on the volume, range, availability, and quality of ophthalmological interventions.This term is formulated based on data analysis of scientific publications and studies [9–14] on the payment system based on diagnostic-related groups (DRG).

The data from numerous publications show that starting from the 1990s, the DRG systems were implemented at the international level [9–14]. This method of payment for inpatient medical services, according to the experience of many countries, made it possible to increase the transparency of the services provided, and payment based on them provided incentives for efficient use of resources [10, 11]. The advantages of the DRG payment system are not only reflected in the increased efficiency and transparency, but also reduced average length of stay [9].

DRG is a relatively scientific and advanced medical payment model recognized globally [11, 12]. In this model, the severity and complexity of disease are compatible, as well as the consumption of medical resources. Disease-based payment is thus achieved. Furthermore, the wide implementation of DRG payment in areas like quality control, cost accounting, and human resource management is realized [13, 14].

The authors considered the term "good practice" in terms of the impact of financing models for ophthalmological care on ensuring the availability and quality of interventions in this area, and not as a "gold standard" or "excellence" of financing models [7].

A definition of "best practice" was explored as "knowledge of what works in specific situations and contexts without using excessive resources to achieve desired outcomes and can be used to develop and implement solutions adapted to similar health problems in other situations and contexts" [15]. The obtained results of the project made it possible to single out separate models of financing ophthalmological services or their components, which can be implemented during reforms of the system of financing ophthalmological care in Ukraine.The article comprises descriptions of methods, results, and arguments to consider while analysing good practices in ophthalmic along with conclusions from our research.

## 2. Methods and materials

The mixed-method research was conducted. Based on legal acts analysis and publicly available data on service utilisation for ophthalmology services we were able to identify thirty-five indicators of the six different ophthalmic diseases listed above for five countries participating in the project. For this article data was used only for Hungary, Poland, and Ukraine. Our analysis covered the period between 2010 and 2020.

The focus of the comparison between countries is, on the one hand, the annual performance at the national level: the number of ambulatory cases or cases treated in one-day surgery or acute inpatient care, as well as access to appropriate ophthalmic services. On the other hand, to compare the regulatory background and its changes within the period analysed. It includes the main characteristics, types, and innovations of reimbursement and payment incentive systems, as well as the regulatory elements of quality assurance. We concentrated on country comparison of 2019 data as it was the last year before the COVID-19 pandemic, but we gathered the latest possible data published for the healthcare sector in each country. We included publications on disease burden financing ophthalmic care in the countries of our scope.

The second phase of our research was to get qualitative information on financing mechanisms assumed to be a good practice so in-depth interviews were run with key stakeholders–healthcare providers and insurance bodies. This altogether allowed us to prepare the final list of good practices in financing ophthalmology services in Poland and Hungary. The last phase of our research was to verify which of the identified good practices in financing mechanisms of ophthalmic services could be implemented in another country. Based on materials from Poland and Hungary on good practices in ophthalmology, Ukrainian researchers developed a separate questionnaire and conducted in-depth interviews among the main stakeholders who ensure the reform, among providers from the public and private sectors and the national procurement agency for medical services regarding the implementation of the identified good practices in Ukraine. Hence, there were two interview guidebooks prepared (S1 and S2 Tables).

It is essential to understand the rationale for recommendations, which were part of our research and widely covered in the summary report from the project [7] for all Visegrad countries and Ukraine. This article is laser focused on features in financing mechanisms only in Poland and Hungary that could be helpful for decision makers in Ukraine and the phase with qualitative research, with findings that have not been published in the summary report [7].

The qualitative research was conducted following the standards for reporting qualitative research [16]. All interviewers had experience in conducting qualitative research. All participants, representatives from healthcare providers and payers, have at least 10 years’ experience in ophthalmology care and knowledge about financing mechanisms in each country of residence. They all agreed voluntarily (informed verbal consent) to participate in interviews and share their perspectives. All interview data was analysed using thematic analysis. Data analysis started after the first interviews were transcribed and checked for accuracy. This was to ensure that the interview guide was adequately eliciting responses on researchers’ experiences and to monitor data saturation. Qualitative data were generated using semi-structured interviews conducted with the participants from Poland and Hungary between June 1^st^ and September 30^th^, 2022, and with the participants from Ukraine between December 1^st^, 2022, and March 30^th^, 2023. Interviews were conducted by three teams (from Poland, Hungary, and Ukraine), and each team consisted of two researchers–interviewers (MT and CD for Hungary; BW and KB for Poland; OS and TY for Ukraine). All interviews took place online using Microsoft Teams. Interviews lasted between 35 minutes and 91 minutes (mean = 64 minutes). The semi-structured approach allowed for interviewers to follow natural flows and avenues of conversation, the interview guide helped reduce potential interviewer bias by ensuring that certain questions were asked and in a comparable way. All interviews were anonymized and reviewed by BW. All interviewers had access to information that could identify the interviewee.

We obtained the resolution from the Committee on Research Ethics of NaUKMA, Registration number: Federal Wide Assurance №00030125. The Committee concluded that the approval of the materials is not required, therefore, the request for examination of the materials was rejected and closed, as the questionnaire for healthcare experts was designed to gather information on their perceptions regarding financing mechanisms in each country based on publicly available information. The study protocol stipulated that no personal health information would be collected. The consent to participate in the interview was provided verbally and was witnessed by two members of the research team from each country. Details about the process of gathering consents were included in the research protocol, which was reviewed by the Ethics Committee.

## 3. Results

The interviews were conducted with fifteen participants (53% female; 47% male), five per country, were interviewed to gather healthcare provider (80%) and payer (20%) perspectives from each country. The analysis of regulatory changes and healthcare utilisation along with in the insights shared during the interviews has led us to the following observations and findings.

### The description of the health care delivery and financing mechanisms

In Poland DRG system (called Jednorodne Grupy Pacjentów) was implemented in 2008. At the same time, financial products “ambulatory DRG” (defined by procedures) were implemented in ambulatory settings (the same no matter the type of specialists). For both types of healthcare services financing was limited to the contract level (defined for each clinic/out-patient specialist). In 2015 the reform called “hospital network” was implemented. Hospitals listed in the hospital network and belonging to "hospital" ambulatory settings receive a global budget one for all outpatient and inpatient clinics) based on services performed one year before (DRG plays as points and is used to settle a budget for next year). The "satellite" ambulatory providers (not being part of any hospital listed in the hospital network system) and hospitals not included in the hospital network system are still financed with an ambulatory/hospital DRG up to a limit defined separately for each type of outpatient/inpatient clinic. As a result, the ophthalmological cases are financed with two different regimes (i.e. DRG used as a payment method or as a point system) depending on being or not on “hospital network” lists. Above it, there are some exceptions–services that are financed by DRG on a fee-for-service basis (i.e. without any limits for providers). These are for example child delivery and cataracts. There is no co-payment for healthcare services in the Polish healthcare system. If a patient wants a higher quality treatment or device, he must cover the whole cost of treatment.

Since 1993 in Hungary the basis of payment mechanism the NHIF has applied the DRGs-based payment scheme (the Hungarian version is Homogén Betegségcsoport: HBCs) both for in-patient and one-day surgery care. For each DRGs, there is a special weight number that expresses the relative cost level as compared to the national average, and it is multiplied by the national equal base rate. For outpatient care since 1993, it has been used the so-called German score system based on the detailed list of activities calculated in scores (points), each activity code has a fixed number of points. Both the ambulance scores (HUF/point) value and the DRGs base rate are predetermined as a national equal tariff announced by the Ministry that is responsible for the Health Affairs (MoH). The MoH announced different ministerial orders that regulate the detailed list of financed out-patient ophthalmology services, including diagnostic examinations (e.g. OTC) and treatment technologies and in-patient services, short-term care distinguishing twenty-five different DRGs (mainly surgeries). In Hungary, in the compulsory health insurance scheme there is no generic co-payment system, just for the pharmaceutical for ophthalmic treatment, the patients have to contribute 10%, 20%, and 45% of the total price. During the COVID-19 pandemic, the abovementioned performance-based payment system was temporarily suspended, but since the 1^st^ of February 2023. The former system was applied again with smaller modifications in the codes and tariffs it is true for the formerly successful cataract waiting list reduction programme that was launched again.

Four periods of changes in the financing mechanisms of the health care system in Ukraine can be identified. The first period took place until 2018 when the financing of the treatment of all ophthalmological cases in the public sector was conducted according to the fixed budget method. The second period began in 2018 and lasted until the first quarter of 2020—the reform of the health care system in Ukraine began. New payment mechanisms were introduced at the level of primary medical care using the capitation method, and the hospital and outpatient sectors, which provided specialized care to the country’s population, were financed based on a fixed budget. The third period began in 2020 and is characterized by the fact that the financing reform was extended to secondary outpatient and inpatient care. Surgical treatments of patients with ophthalmological cases were included in the package of medical guarantees "Surgery for adults and children in hospital". The fourth period started in 2021 when the principles of financing by diagnostic and related groups (DRGs) began to be applied, and reforming according to this method of payment is still taking place. In 2022, ophthalmological cases will be included in the package of medical guarantees "Surgery for adults and children within one-day hospitalization".

It is very difficult to compare the prices of healthcare services between Hungary and Poland, as they differ between countries by ICD-10 (disease types) and/or ICD-9 codes even if they are defined by DRGs. In Ukraine, healthcare services only started to be determined by DRGs in 2021, i.e. specialized medical services were paid according to the global budget, considering adjustment factors. From 2022, payment for healthcare services is conducted as a combination of the global budget and the global rate and payment for a treated case of DRGs (S3 Table).

The descriptions of the health care delivery and financing ophthalmology services in Hungary and Poland have led us to the conclusion that there are significant differences across these countries. The differences exist although both countries have implemented DRG-based systems for hospital care for a long time. The comparison of practices in each country allowed us to identify these differences and define good practices in treatments for cataract, glaucoma, AMD, DME, cornea transplantations, and vitrectomy. As was found in the summary report, there is no good practice in financing leading to increase access to all services in ophthalmology and financial incentives differ not only across countries but also across diseases [7].

### Identification of practices of health care services and financing of ophthalmology

Cataract is and will continue to be, the most frequent treatment provided in ophthalmology care. As our populations are aging, ensuring access to treat the problem of visual impairment when needed will become essential. The major difference identified during our research pertains to the treatment scheme. In Hungary cataract surgeries can be financed within the ambulatory care, while in Poland only in hospital care. This now contributes ’only’ to the higher costs of care, as the mechanism of a financial penalty (a 10% decrease in DRG price) in the case of one-day treatments lower than 80% leads to a sharp increase in short hospitalizations. With 98% of daily cases (Zdrowe dane. Choroby narządu wzroku), Poland does not differ much from the ambulatory treatment scheme provided in Hungary.

The main barrier to access treatment is also limited (put by third-party payers on healthcare providers) in financing the number of cataract surgeries. No limits on volumes introduced in Poland in 2018 allowed to treat patients without unnecessary delay. Together with separating the payment for qualification visits (with specific medical criteria for cataract surgery) it increased in number of treatments (S4 Table) and reducing significantly the waiting time for treatment (from 489 days in 2016 to month to 127 days in 2020 in Poland; Zdrowe dane. Choroby narządu wzroku). The average waiting time for cataract surgery was 104 days in 2014 and 39 days in 2019 after a few years of a conscious continuous waiting time reduction program organized and financed by the NHIF in Hungary [24].

The second disease that was explored by the project team members in each country was glaucoma. During our team discussions and interviews with healthcare providers, we discovered that glaucoma is mostly treated with the use of pharmacotherapy. Therefore, ensuring access to many medicines that correspond to the wide variety of clinical conditions of patients diagnosed with glaucoma was crucial. The wide list of reimbursed drugs allows matching the treatment with health needs while being affordable for patients. According to a Polish medical doctor who works in an ambulatory care facility with the public-payer contract and private facilities: *There is great access to modern drug treatments*, *with huge public funding*, *especially in the age group 75+* (P2).

For glaucoma and vitrectomy also, financial limits were released in Poland for ambulatory as well as for surgical care. During interviews, they were pointed out as “a wish good practice” (*Distinguishing the consultation- qualification for treatment*, *cataract surgery*, *and follow-up consultation was the key to providing access with the right financing*, *including removing the limits for financing surgeries*. *There is no data about waiting time for glaucoma treatment*. *There are waiting lists for vitrectomy*. *These procedures probably require removing limits too*. *(…) Removing limitations in financing medical procedures in ophthalmology could help to increase access to services*. (P3). *The requirements and proper financing would have to be adjusted to move more treatments to 1-day hospitalizations*. *Cataract*, *glaucoma*, *and vitrectomy could be mostly conducted during the 1day stay* (P5).

AMD was another disease that was analysed. Increasing access to health care can be achieved by adjusting the financial product to the complexity of the procedure. The greatest shift in volumes was observed in Poland, where in 2018 introduction of the so-called “drug programme” allowed the gradual transition from inpatient hospitalizations to outpatient care (from 9,790 in 2015 to 27,787 in 2020, Zdrowe dane. Choroby narządu wzroku). *It [the program] is one of the few things that works*. *It is an example of how the pricing influences the market*: *the better pricing pushed providers to buy medical equipment and equipment allowed to diagnose the patients and this resulted in higher access to treatment* (P6). In Hungary this programme is defined as an ambulatory treatment provided by the hospital so it realised capacity resources in hospitals leading to a sharp increase in the number of people treated (from 4,500 in 2014 to 19,300 in 2019 in Hungary’s NHIF financial database), the payment method was the same than for cataracts a special DRGs code was set up exactly for AMD neovascularisation for relatively low tariffs (RW: 0,15122), appr. 80 EUR/case without volume limit for the hospitals.

When analysing the financial mechanism for treatment for DME, wide coverage of diagnostics in Hungary stood up as good practice both increasing access and quality of care respectively (*The diabetic ambulance service (the network of diabetic care centres) itself are quite well organized*, *who are already diagnosed can receive appropriate care*, *yearly control of the typical complications (among them the blindness)*, *however*, *it should be recommendable to launch further national early detection campaign of DME* (P9). Regarding DME following diagnostic services are financed: detailed anamnesis and basic examination: eye movement examination, digital eye pressure estimation, slit lamp examination, a subjective determination of refraction, ophthalmoscopy, blood pressure, blood lipids, gonioscopy, fluorescein angiography, OCT optical coherence tomography. A good practice influences many treatments in financing healthcare services, where it is possible, in outpatient care. In Poland, the introduction of a so-called drug programme for DME as outpatient treatment (realized by high-quality hospitals) lead to allow to help these patients most efficiently (..)*the program is developing*, *and more and more patients are being treated so access to care is improving* (P4).

Highly complicated and relatively low frequency realized procedures should be subject to centralisation (i.e. performed only in a high-quality health centre). In ophthalmology, such procedure is cornea transplantations. Acquiring organs, tissues, and cells for these procedures are the top barriers to access. Functioning tissue banks organising a sufficient number of transplants is the key to covering the demand for treatment. The other thing is payment mechanisms. If the cost of the cornea is the same for all providers DRG must cover the whole cost of the cornea and the cost of the medical procedure, as in Hungary (*The DRGs on cornea transplant cover the following interventions and activities*: *costs of extraction of donor cornea*, *detailed anamnesis and basic examination*: *eye movement examination*, *digital eye pressure estimation*, *slit-lamp examination*, *subjective determination of refraction*, *ophthalmoscopy* (P10). In Poland, there are tissue banks financed from public sources (i.e. the cornea is given for free to the provider) and tissue banks that do not receive public financing, so the cost of the cornea has to be covered by a healthcare provider. Separating the payments for cornea from the payments for transplantations was an important step towards increasing access to this service by covering costs for those health providers that procure cornea from commercial cell and tissue banks if they do not receive cornea from the public ones. Such financial incentives were implemented in Poland. *We should conduct about five thousand surgeries per year*, *we do about 1200*. *The longer patients wait*, *the more invasive transplantation*, *hence more complications*, *and higher costs for everyone*. *There are mechanisms to gather cornea and incentives for gathering cornea* (P4).

Financing schemes can also influence the quality of treatment. In this type of action, some incentives were identified the same for several types of ophthalmological diseases. The most common were procedures to centralise care in high-quality healthcare centres. This was a case for special glaucoma surgery and vitrectomy in Hungary (provided by the four Medical Universities, and in some other county hospitals), AMD and DME in Poland (separate contracts for hospitals performing certain conditions), and cornea transplant in Hungary and Poland. (*For some procedures*, *like for glaucoma*, *it would be good to have centralization*, *for those that are rather rare*, *not in thousands*, *so that we have highly specialized centres to deal with these rare or more complicated cases*. [corneal transplantation] *It should not be centralized because we need too many transplantations* (P4). *Several factors would be highlighted for the concentration of special and very expensive treatments and surgeries*: *the composition of the team*, *the improving organizational culture*, *and team building*, *the learning curve of physicians*, *and economies of scale all can contribute to a better cost-effectiveness and higher outcome* (P9).

For cataract treatment, it was stated that mechanisms ensuring flexibility of payment are good practices for increasing better access to treatment as they are the best way of matching patient needs (*The ideal patient pathway*: *to detect the problem at a lower level*, *in any secondary care provider let’s be in a smaller city*, *county hospital*, *or University Clinic outpatient department*. *After receiving the referral to the surgery centre the patient must be diagnosed*, *and examined to the patient should be thoroughly*, *to decide about the best available and appropriate lens*, *soon there be a discussion and agreement with the patient about the best option and its financing if it needs additional private payment (co-payment)* (P8). In both analysed countries we found some mechanisms of that kind. In Poland, the price for DRG groups is adjusted by a factor of 1.25 when using toric lenses or iridial lenses (implemented in 2018). This 25% additional payment is aimed at covering the difference in costs between “classic” lenses (covered in DRG price) and toric/iridial ones. In Hungary, there has been introduced a new DRG group (since 2014) dedicated only to the costs of surgery. Hospital material, hotel costs, and human resource costs are reimbursed by the public third-party payer (NHIF), while the cost of a new lens implant is paid directly by the patient. So, the patient can choose between a whole public payment (cost of the lens included in DRG price) or part public payment (cos of the lens is covered by out-of-pocket payment).

Quality indicators measured while providing healthcare services and making them publicly available result in better healthcare outcomes. In Poland, such indicators were added to the DRG scheme. When reporting cataract DRG healthcare providers are obligated to report an assessment of surgeries. Three indicators are reported with DRG (to each patient case): posterior capsule rupture, endophthalmitis, and change in visual acuity. Especially vision impairment measurement (before and after) surgery appeared to be a patient-choice sensitive indicator.

Also, the definition of financial products can influence the treatment quality. The most important practices identified by our research team were payment for each injection separately and financial products for evaluation of achieved outcomes. Payment for each injection imposes elasticity for treatment conditions for each patient. It is implemented in Poland and Hungary for AMD and in Poland for DME. The change in financing with the fee-for-service payments in 2021 can contribute to greater flexibility in adjusting the treatment scheme to patients’ needs and coverage of providers’ costs linked to the treatment. It also enables greater monitoring and keeping up with the clinical excellence guidelines. In Poland, the quality of care was also assured via new financial products to cover the cost of evaluation of achieved health outcomes during a follow-up consultation after the end of treatment.

### Good practices in financing ophthalmology care to be proposed for implementation in Ukraine

The analysis of good practices of Poland and Hungary and the expert opinion of ophthalmologist-surgeons, representatives of the National Health Service as a purchaser of services, and the heads of health care facilities from the public sector in Ukraine showed that the best practice for Ukraine among those proposed by this study, which can be implemented into routine practice at the moment, is separate payment for lenses for the treatment of cataracts. Experts consider separate payment or even co-payment for standard or more individual (rare) lenses as good practice to adopt. The Programme of Medical Guarantees covers most costs, including diagnostics, consumables, surgery, and basic lenses. One of the experts noted the following: *These are very rare lenses*, *so only co-payment or even full payment for lenses because the state cannot cover all lenses*, *especially such individual ones* (P11). The plans for 2023–2024 include a review of pricing and allocation of individual services or medical products for which the patient will have to pay separately: *Right now*, *we are preparing a more detailed list*, *which will*, *as it were*, *break down the general services to understand that we cannot now fully pay for Programme of the Medical Guarantees*. *These services will be included in a separate Cabinet of Ministries Decree*, *which will be supplemented with a list of paid services*. *It will be clearly understood what the state pays for and what it absolutely does not cover by state budget* (P14).

The proposed practices for cataract, which relate to financing under the DRG, quality indicators for DRG, and limits, were classified as good practices and partially supported by Ukrainian experts, since the financing under the DRG is only being implemented in Ukraine, it is difficult for experts to predict what changes will happen soon since these processes depend on the adoption of relevant legislation and the introduction of relevant changes in financing system. Thus, it was difficult for the experts to answer about good practices such as a decrease in DRG payment in case of a low share of one-day treatment, because two funding mechanisms are currently being implemented, one-day treatment under the Programme of Medical Guarantees, and the DRG financing system: *Currently*, *all ophthalmology is carried out in outpatient settings*, *or it is 1-day surgery*. *In rare cases*, *the patient may be hospitalized* (P12). Experts do not know how the healthcare system will be related to DGR soon.

However, different package prices for three lens types as a good practice for cataract was not supported by experts: *Due to the high specificity*, *a very small percentage of toric and aniridia lenses are used*, *some fractions of a percent*, *so no*, *it is better to cover average used lenses*. *The specificity of lenses for these patients is very high*, *there are very few of them*…*These lenses are very specific*, *sometimes even individual*, *and their production can be more expensive than the expected social result*, *so I don’t think that covering from the budget is appropriate to these types of lenses* (P13).

Unequivocal support from the ophthalmological community was received for the good practice of Poland and Hungary in the maximum expansion of the National list of medications (both original medicines and generics) for the treatment of glaucoma. Experts from Ukraine also consider that *it is necessary to expand the "Affordable Medicines" reimbursement program and add absolutely all medicines of various classes used for the treatment of glaucoma* (P13). This is justified by the fact that *glaucoma as a disease has a significant burden on the state because if it is detected or started treatment too late*, *it can lead to absolute blindness*, *and therefore increase the level of disability from glaucoma* (P12). The experts supported a good practice as wide coverage of diagnostics for DME and ADM: *Screening programs are needed*, *because these are the diseases that*, *in suppressed stages*, *can carry a social burden for the state and increase disability due to blindness* (P14). *‘There is no systematic screening of the early stage of AMD*. *Early diagnosis and early treatment would result in a better outcome* (P9). Overall, it can be said that in Hungary there is no national protocol for the organization of early detection and effective treatment of AMD among the rapidly aging population. It was also said that *primary care prevention in ophthalmology is usually more expensive than treatment; it is very expensive to cover from the state budget*, *but screening programs are possible to implement* (P10). AMD and DME screening programs are needed because *these diseases can carry a social burden for the state and increase disability due to blindness*. *International standards of treatment are supported in the country*, *including following the rules of primary diagnosis*, *but perhaps the government and the Ministry of Health should pay more attention to screenings* (P15).

Financial products for evaluation of achieved outcomes and payment for each injection, including the biological treatment anti-VEGF, separately were recognized as good AMD and DME practices that should be implemented in Ukraine after the adoption of necessary legislation. Experts noted that *medications for the treatment of AMD and DME are expensive*, *so none of them are sure whether patients will be able to cover all injections out of pocket*, *and whether the state budget can cover everything*. *Perhaps it is worth periodically launching local state programs*, *within which such medicines would be purchased in regions or state clinics with the highest demand* (P14). Also, it is necessary “*to expand the state list of medicines due to their high cost to increase access to services* (P12). As a result, the implementation of this good practice should be postponed.

Cornea transplantation is currently not included in a separate package of the Programme of Medical Guarantees, because *such surgery is still very difficult to cover fully* (P15). However, it is conducted within the "Surgery for adults and children in hospital" package, as it is a high-tech surgery. This package covers all examinations, surgery, and partial consumables. Transplantation in Ukraine is already performed only in specialized centres, particularly in Dnipro, Odesa, and Kyiv, and *it cannot be otherwise*, *because the patient must be prepared by all standards* (P15). Experts found the functioning of tissue banks and separate payments for procedures and cornea as a good practice, but at the legislative level, there is currently no permission to take corneas from commercial cells and tissue banks. There is permission both to use only state banks, but *the quality of the cornea is not high* (P13), and to conduct transplantation from a posthumous donor. Of course, *functioning tissue banks to have sufficient transplants is a good practice*, *but Ukraine must first develop a legislative framework and all the conditions for conducting high-quality corneal transplantation* (P12). Some experts said that there is support for implementing a separate package of medical guarantees for transplants, but the circumstances in Ukraine currently do not provide a clear framework for realization.

The system of providing medical services is currently constructed in Ukraine in such a way that surgery for almost all nosologies is performed at the inpatient level or as a one-day hospitalization. Therefore, experts from Ukraine did not support dedicated centres for surgeries of all nosologies as a good practice to be implemented in Ukraine. To implement such a practice, it is necessary to change the approach and system from the legislative level, which is currently difficult and almost impossible in the current conditions.

During the interviews with experts, it was revealed that it would be a good practice for Ukraine to perform vitrectomy surgery in specialized institutions to increase quality, which also provides greater opportunities. At the same time, experts doubted that such good practice will improve the quality of vitrectomy services in Ukraine. It is commented by the following: *This is a good practice*, *but in our country*, *everything is regulated by certain laws and decrees* (P12). Implementing these good practices requires significant legal work upfront.

## 4. Discussion

Several definitions of good practice were found in the literature on health economics and financing healthcare services. In the Polish literature attempts to define good practice in financing healthcare services are brought on when researchers discuss healthcare financing reform, changes in healthcare financing in Poland, and good practices in healthcare financing in Poland and the European Union [17–19]. They provide insights into the challenges and opportunities in healthcare financing in Poland and highlight the importance of efficient and effective resource allocation, universal coverage, and sustainability in healthcare financing. In Hungary, good practices were consciously defined, collected, and disseminated in the frame of an EU funded project focusing on patient safety and effectiveness [20, 21]. It is also mentioned in the context of the effectiveness of special treatments for select diseases in ophthalmology [22]. In Ukraine, similarly to Poland and Hungary, there is no fixed term or definition of “good practice” in legal acts pertaining to financing health care services. We needed the common denominator for Hungary, Poland, and Ukraine therefore we used a very broad definition that comprises all financial mechanisms that impact the volume or quality of ophthalmology care.

There are ample reasons why a financial mechanism may be perceived to be a good practice in one country and cannot be defined as a good practice in another country. These reasons can be grouped into four categories: geographic/regional; cultural/historic/regulatory background; financing/organisational (centralization or decentralization); and infrastructure/workforce-related (availability of facilities/doctors) [23].

Geographic factors can influence the effectiveness of a financial mechanism in different countries. For example, in a country with a large rural population or regions with limited access to healthcare facilities, a decentralized financial mechanism may be more suitable. It allows for greater flexibility and localized decision-making to address regional disparities in healthcare access [24, 25]. On the other hand, in densely populated urban areas, a centralized financial mechanism might be more efficient. It can streamline resource allocation, coordination, and service delivery.

Cultural, historic, and regulatory factors can shape the perception of a financial mechanism as a good practice. These factors include:

- cultural attitudes towards healthcare—different cultures may have varying expectations, preferences, and attitudes towards healthcare financing and delivery [26]. A financial mechanism that aligns with cultural norms and values is more likely to be perceived as effective [27].
- Historic context and healthcare system development—countries with different historical trajectories may have unique healthcare systems and financing mechanisms that have evolved [26]. The compatibility of a financial mechanism with the existing system plays a significant role in its perceived effectiveness [28].
- Regulatory frameworks—regulatory policies, such as healthcare regulations and insurance laws, vary across countries. A financial mechanism that aligns with existing regulations and legal frameworks is more likely to be considered a good practice [29].

The financing and organizational aspects of a healthcare system can impact the suitability of a financial mechanism [30]. Given the results of our research, the key considerations include:

1. Funding availability and allocation. Countries with various levels of financial resources like Hungary, Poland, may require different financial mechanisms to ensure sustainable and equitable healthcare financing.
2. Centralization or decentralization. The degree of centralization or decentralization in a healthcare system, like in Poland and Hungary, can influence the effectiveness of a financial mechanism. Centralized systems might prioritize efficiency and resource allocation, while decentralized systems, may focus on local autonomy and responsiveness.
3. Governance and administrative structures. The organizational structure of healthcare systems, such as the presence of a single-payer system (Poland), multiple insurance providers, or a mix of public and private entities (like in Hungary), can affect the implementation and success of a financial mechanism.

Finally, infrastructure and workforce considerations can impact the viability of a financial mechanism [31]. Factors to consider include availability of healthcare facilities and healthcare professionals. Countries with disparate availability or distribution of healthcare facilities may require different financial mechanisms to ensure equal access to care. Similar observation can be made about healthcare workforce—the availability and distribution of healthcare professionals, such as doctors and specialists, can influence the effectiveness of a financial mechanism. A mechanism that accounts for workforce shortages or maldistribution is more likely to be considered a good practice.

It is important to note that these categories are not exhaustive, and multiple factors can interplay within each category. Additionally, each country’s unique circumstances and priorities contribute to the perception of a financial mechanism as a good practice [31]. Therefore, when evaluating the suitability of a financial mechanism, it is crucial to consider the specific context and needs of each country. During the interviews with experts from Hungary, Poland, and Ukraine we identified three main differences in perceptions of good practices and the rationale for implementing them in Ukraine. After conducting the interviews, three positions regarding the application of good practices in Ukraine were revealed. In general, all practices are recognized as good, but there are certain barriers and reasons for the implementation of each of them currently in Ukraine [29, 32, 33].

The first category of perception fully supports good practices due to their importance in Ukraine. This is because an appropriate legal framework has already been developed, financing mechanisms have been introduced, and the implementation of DRGs (Diagnosis-Related Groups) has been expanded, which will be conducted in the near future.

The second category of perception supports good practices in general. However, due to uncertain financial possibilities, the implementation of these practices is currently in question. This is because the country is facing a tough economic situation, and it is challenging to predict the availability of funds in state and local budgets.

The third category of perception involves good practices that are currently challenging to implement. This is due to an undeveloped regulatory and legislative framework, as well as an incomplete reform process. These practices are not currently supported by experts for implementation. However, it is possible for Ukraine to revisit these practices once the situation stabilizes and the hostilities come to an end.

Findings from the interviews in Ukraine prompted us to re-examine current developments in financing ophthalmic care. One of the interviewees brought our attention to a good practice in Poland, which involves removing limitations in financing treatments for glaucoma and vitrectomy.

We identified several limitations during our research. The limitation of this study from the side of Ukraine is that there were no changes in healthcare financing mechanisms until 2017. The changes that began to take place after led to the transition of ophthalmology more into the private sector. However, since 2020, significant changes have begun in the financing of secondary specialized care in ophthalmology. That is, since 2020, there is still a transition to a new financing model, which includes financing under the DRGs. In Poland, the most important challenge was missing data for some years in reporting for some procedures and scant information for ambulatory care services in ophthalmology [34]. Also, datasets with aggregated data points about the utilisation of services and changes in publishing practices posed a challenge in data analysis. Finally, we came across a particularly important limitation in descriptions of financial products, including enlisting ICD-9 and ICD-10 medical procedures or lack thereof, hence it was impossible to conduct international comparisons for all diseases included in our research as primarily designed. These limitations were also identified in other studies [35, 36]. Using qualitative research methods aimed to achieve the research objective despite the above limitations.

## 5. Conclusions

For all these three countries with populations that heavily depend on healthcare services funded from public sources, health equity needs to be embedded in the national health policies. Financing mechanisms in healthcare are subject to constant changes and therefore monitoring and evaluation of interventions is a must-have to assess effectiveness and impact on access to healthcare services in ophthalmology. We have also identified additional research areas such as measuring the quality of care in our countries or creating a flexible financing scheme that can incorporate new treatment methods, devices, and surgical techniques.

It is difficult to define one financial mechanism that fits all needs in countries of our interest, we believe that it is possible to name practices that could be used to increase the volume of services as well as to define a mechanism to effect on the quality of care. Our research findings suggest that decision-makers should focus not only on drafting interventions that target to increase service volume reducing waiting time but also those that enable monitoring of achieved health outcomes and incentivise increasing quality of care in ophthalmology. Therefore, all partners are interested in the implementation of best practices and experience, because of the most effective use of public funds and resources; moreover, the healthcare system in Ukraine is currently undergoing reform.

Based on the Hungarian experience to keep the flexibility of activity and reimbursement code system in different settings like outpatient care, inpatient care, and one-day surgery as well as incorporating new (innovative) technologies as soon as possible—would be an essential lesson for Ukraine healthcare system.

In Poland, it has been essential to remove financial barriers such as limitations for financing ophthalmic care. These barriers were removed for cataract glaucoma and vitrectomy, but they are still existing in other eye diseases. It is also important to monitor the availability of resources, including ensuring the training of a sufficient number of specialists and measuring the quality of care—might offer good regulatory direction to Ukrainian decision-makers.

The results of this research are significant for Ukraine, because the health care system is currently at the stage of reform, and Ukrainian decision-makers need to focus on good practices to improve the system. Therefore, Ukraine is currently looking for optimal good practices for the country, and it is important to consider the experience of other countries, which can be considered through the prism of the healthcare financing system in the country.

## Supporting information

S1 Table Interview guidebook for participants based in Poland and Hungary. (DOCX)

S2 Table Interview guidebook for participants in Ukraine. (DOCX)

S3 Table Financial mechanisms for ophthalmology treatment (2019). (DOCX)

S4 Table Cataract volumes (% change to the previous year) (in thousands). (DOCX)

## References

[1] World Health Organization. World Report on Vision. Geneva, Switzerland: World Health Organization; October 8, 2019. https://www.who.int/publications/i/item/9789241516570. Accessed 6 Jan 2023

[2] Friedman DS, Wolfs RC, O Colmain BJ, et al. (2004). Prevalence of open-angle glaucoma among adults in the United States. *Arch Ophthalmol*, 122(4), 532–538. doi: 10.1001/archopht.122.4.532 15078671 PMC2798086

[3] Gorin MB, Breitner JC, De Jong PT, et al. The genetics of age-related macular degeneration. *Mol Vis*. 1999; 5:29. Published 1999 Nov 3. 10562653

[4] Yau JW, Rogers SL, Kawasaki R, et al. Global prevalence, and major risk factors of diabetic retinopathy. *Diabetes Care*. 2012;35(3):556–564. doi: 10.2337/dc11-1909 22301125 PMC3322721

[5] Klein R., Rejdak R, Juenemann AG, Natarajan S. Posterior Segment Ocular Trauma: Timing and Indications for Vitrectomy. *J Ophthalmol*. 2017; 2017:5250924. doi: 10.1155/2017/5250924 29214076 PMC5682070

[6] Самооцінка населенням стану здоров’я та рівня доступності окремих видів медичної допомоги у 2019 році. Статистичний збірник. Державна служба статистики України. К., 2020 ДЕРЖАВНИЙ КОМІТЕТ СТАТИСТИКИ УКРАЇНИ (ukrstat.gov.ua) Accessed 6 Jan 2023. [Population’s self-perceived of health status and availability of selected types of medical aid in 2019. Statistical collection. State Statistics Service of Ukraine. K., 2020 State Committee of Statistics of Ukraine (ukrstat.gov.ua) Accessed 6 Jan 2023]

[7] Wieckowska B, Byszek K, Rekas M, Yurochko T, Shevchenko M, Skrypnikova O, et al., The summary report on good practices in financing healthcare services in ophthalmology, May 2023 https://www.sgh.waw.pl/kes/sites/kes/files/2023-06/VISEGRAD-GRANT-PROJECT-SUMMARY-REPORT.pdf Accessed 30 June 2023

[8] World Health Organization. Guide for documenting and sharing “Best Practices” in Health Programmes. Regional Office for Africa Brazzaville. 2008. https://www.afro.who.int/sites/default/files/2017-06/Guide_for_documenting_and_Sharing_Best_Practice_-_english_0.pdf. Accessed 17 Feb 2023

[9] Goldfield N. (2010). The evolution of diagnosis-related groups (DRGs): from its beginnings in case-mix and resource use theory, to its implementation for payment and now for its current utilization for quality within and outside the hospital. *Quality management in health care*, 19(1), 3–16. doi: 10.1097/QMH.0b013e3181ccbcc3 20042929

[10] Mihailovic N., Kocic S., & Jakovljevic M. (2016). Review of Diagnosis-Related Group-Based Financing of Hospital Care. *Health services research and managerial epidemiology*, 3, 2333392816647892. doi: 10.1177/2333392816647892 28462278 PMC5266471

[11] Ma Y, & Wang W. (2021). The impact of diagnosis related group payment on the performance of public hospitals. *American journal of translational research*, 13(6), 6796–6801. 34306429 PMC8290653

[12] Montefiori M, Pasquarella M, & Petralia P. (2020). The effectiveness of the neonatal diagnosis-related group scheme. *PloS one*, 15(8), e0236695. doi: 10.1371/journal.pone.0236695 32785282 PMC7423098

[13] BiørnE Hagen TP, Iversen T, Magnussen J. (2010). How different are hospitals’ responses to a financial reform? The impact on efficiency of activity-based financing. *Health care management science*, 13(1), 1–16. doi: 10.1007/s10729-009-9106-y 20402278

[14] Dimitropoulos V, Yeend T, Zhou Q, McAlister S, Navakatikyan M, Hoyle P, et al. (2019). A new clinical complexity model for the Australian Refined Diagnosis Related Groups. *Health policy (Amsterdam*, *Netherlands)*, 123(11), 1049–1052. doi: 10.1016/j.healthpol.2019.08.012 31506190

[15] UNAIDS best practice collection.1999. https://digitallibrary.un.org/record/371211?ln=en. Accessed 17 Mar 2023.

[16] O’Brien BC, Harris IB, Beckman TJ, Reed DA, & Cook DA (2014). Standards for reporting qualitative research: a synthesis of recommendations. Academic medicine: *Journal of the Association of American Medical Colleges*, 89(9), 1245–1251. doi: 10.1097/ACM.0000000000000388 24979285

[17] Czech M, Głód M (2021). Reforma finansowania opieki zdrowotnej w Polsce–cele, wyzwania, instrumenty. [Healthcare financing reform in Poland–goals, challenges, instruments.] *Medycyna Praktyczna*, 4(145), 34–42. https://www.mp.pl/pacjent/kompendium-wiedzy/reforma-finansowania-opieki-zdrowotnej-w-polsce-cele-wyzwania-instrumenty

[18] Kocot E. (2019). Zmiany w finansowaniu opieki zdrowotnej w Polsce w kontekście reformy systemu ochrony zdrowia. [Changes in healthcare financing in Poland in the context of healthcare system reform.] *Pielęgniarstwo i Zdrowie Publiczne*, 9(3), 115–121. doi: 10.2478/pielzop-2019-0022

[19] Kowalska-Bobko I, Domagała A. (2018). Dobra praktyka finansowania opieki zdrowotnej w Polsce i w Unii Europejskiej. [Good practices in healthcare financing in Poland and in the European Union.] *Problemy Zarządzania*, 16(1), 15–27.

[20] Semmelweis University, 2020 https://jogyakorlatok.betegbiztonsag.info/#/project Accessed 6 Jan 2023.

[21] Az egészségügyi ellátórendszer módszertani fejlesztése, EU funded project: 2017–2020, EFOP-1.8.04-VEKOP-17-2017-00001 Egészségügyi jó gyakorlatok online katalógusa. (Catalog of best practices in HealthCare)

[22] Tóth G, Nagy Z, Németh J. Model-based economic burden of diabetic retinopathy in Hungary (A cukorbetegség szemészeti szövődményeinek modellalapú költségterhe Magyarországon.) Orvosi Hetilap. 2021. 162/8.:298–305. doi: 10.1556/650.2021.32031 33611265

[23] Solomon SD, Shoge RY, Ervin AM, et al. Improving Access to Eye Care: A Systematic Review of the Literature. Ophthalmology. 2022;129(10): e114–e126. doi: 10.1016/j.ophtha.2022.07.012 36058739

[24] Pónusz R., Boncz I, Kovács D, Csonka D, Gazsó T, Molics B, et al. Analysis of the medical composition, utilization indicators and territorial distribution of the Hungarian waiting list reduction program in the period 2015–2018. (A magyarországi várólista-csökkentési program orvosszakmai összetételének, igénybevételi mutatóinak és területi megoszlásának elemzése 2015–2018 időszakában.) LAM 2022; 32(3):121–131. doi: 10.33616/lam.32.011

[25] Pónusz R, Endrei D., Kovács D, Csutak A, Boncz I. (2022) The place and role of one-day surgery in reducing waiting list for cataract surgeries (Az egynapos sebészet helye és szerepe a szürkehályog műtét várólista csökkentésében). IME, 2022; 21(4):3–10.

[26] Rogers L, De Brún A & McAuliffe E. Defining and assessing context in healthcare implementation studies: a systematic review. *BMC Health Serv Res* 20, 591 (2020). doi: 10.1186/s12913-020-05212-7 32600396 PMC7322847

[27] Zurynski Y, Ludlow K, Testa L, et al. Built to last? Barriers and facilitators of healthcare program sustainability: a systematic integrative review. *Implementation Sci*. 2023;18:62. doi: 10.1186/s13012-023-01315-x 37957669 PMC10641997

[28] Li SA, Jeffs L, Barwick M, et al. Organizational contextual features that influence the implementation of evidence-based practices across healthcare settings: a systematic integrative review. *Syst Rev*. 2018;7:72. doi: 10.1186/s13643-018-0734-5 29729669 PMC5936626

[29] Klaic M, Kapp S, Hudson P, et al. Implementability of healthcare interventions: an overview of reviews and development of a conceptual framework. *Implementation Sci*. 2022;17:10. Available from: doi: 10.1186/s13012-021-01171-7 35086538 PMC8793098

[30] Han X, Chen Y, Gordon I, et al. A Systematic Review of Clinical Practice Guidelines for Age-related Macular Degeneration. *Ophthalmic Epidemiol*. 2023;30(3):213–220. doi: 10.1080/09286586.2022.2059812 35417274

[31] World Health Organization. A Guide to Identifying and Documenting Best Practices in Family Planning Programmes. 2017. https://apps.who.int/iris/bitstream/handle/10665/254748/9789290233534-eng.pdf. Accessed 17 Feb 2023.

[32] On state financial guarantees of medical care for the population: Law of Ukraine 19.10.2017 № 2168-VIII (Про державні фінансові гарантії медичного обслуговування населення: Закон України від 19.10.2017 № 2168-VIII). https://zakon.rada.gov.ua/laws/card/2168-19

[33] Population’s self- perceived of health status and availability of selected types of medical aid (Самооцінка населенням стану здоров’я та рівня доступності окремих видів медичної допомоги у 2019 році). Statistical Bulletin. State Statistics Service of Ukraine. K., 2020 State Committee of Statistics of Ukraine. https://ukrstat.gov.ua/druk/publicat/kat_u/2020/zb/03/snsz_med_2019.pdf. Accessed 17 Feb 2023

[34] Zdrowe dane. Choroby narządu wzroku, Centrum eZdrowia, https://ezdrowie.gov.pl/portal/home/badania-i-dane/zdrowe-dane/monitorowanie/choroby-narzadu-wzroku Accessed 7 May 2023.

[35] Teo ZL, Tham YC, Yu M, et al. Global Prevalence of Diabetic Retinopathy and Projection of Burden through 2045: Systematic Review and Meta-analysis. *Ophthalmology*. 2021;128(11):1580–1591. doi: 10.1016/j.ophtha.2021.04.027 33940045

[36] Tham YC, Li X, Wong TY, Quigley HA, Aung T, Cheng CY. Global prevalence of glaucoma and projections of glaucoma burden through 2040: a systematic review and meta-analysis. Ophthalmology. 2014;121(11):2081–2090. doi: 10.1016/j.ophtha.2014.05.013 24974815

