# Supplementary material for: How much can we learn from each other? Polish and Hungarian good practices in financing ophthalmology care as a proposal for implementation in Ukraine
Source: PLoS One. 2024 Jul 9;19(7):e0306562. doi: 10.1371/journal.pone.0306562 (PMC11232999; doi:10.1371/journal.pone.0306562)
Supplement: S1 Table — (DOCX) [file pone.0306562.s001.docx]

**S1 Table. Interview guidebook for participants based in Poland and Hungary**

1. What is your experience with ophthalmic care services, and in financing medical procedures?
2. How would you assess access to ophthalmic care services in general?
3. Barriers
4. What barriers do you see related to the method of financing affecting the availability and quality of ophthalmic services?

|  | Barriers to access | Barriers to high quality of care |
| --- | --- | --- |
| Cataract |  |  |
| Glaucoma |  |  |
| Vitrectomy |  |  |
| Corneal Transplant |  |  |
| AMD |  |  |
| DME |  |  |

1. How to remove the barriers?

|  | Barriers to access | Barriers to high quality of care |
| --- | --- | --- |
| Cataract |  |  |
| Glaucoma |  |  |
| Vitrectomy |  |  |
| Corneal Transplant |  |  |
| AMD |  |  |
| DME |  |  |

1. Screening* patients for diseases (*preventative care, early detection, diagnosing, any dedicated health program or financial product to screen patients against the diseases, covered from the public funding, either local or central funding)

|  | In place (Y/N) | Is it effective? | How screening should be organized / financed |
| --- | --- | --- | --- |
| Cataract |  |  |  |
| Glaucoma |  |  |  |
|  |  |  |  |
| Corneal transplant |  |  |  |
| AMD |  |  |  |
| DME |  |  |  |

1. Ambulatory care – conservative treatment

What are the practices in the pharmacotherapy? What are the modern therapies? Are they covered by the pubic funding?

1. Ambulatory care – 1-day surgeries/treatments

Are the tariff costs sufficient to cover costs of service providers? Do the rates set by the funding party (insurance funds, a public budget, etc.) cover the costs of providing medical care to ophthalmic patients?

*In many countries, ophthalmic services are available in the form of outpatient / one day clinics, e.g. cataract or vitrectomy. Does the need to implement them under a hospital contract (which is associated with higher costs) affect the availability of services? In other words, if we allowed this type of solution to be public financing, would the availability increase? How about the quality?*

|  | Possibility of ambulatory / 1-day surgery facilities instead of in a hospital |
| --- | --- |
| Cataract |  |
| Glaucoma |  |
| Vitrectomy |  |
| Corneal transplant |  |
| AMD |  |
| DME |  |

1. Ambulatory care – treatments with laser - how do you evaluate access to laser treatments in outpatient health care from public funds, where 1 is limited funding, 5 no limitations in financing this type of services. Please comment / explanation in case of limited financing

| *Laser treatment* | PL | HU | UA* |
| --- | --- | --- | --- |
| Laser capsulotomy (cataract) |  |  |  |
| Laser iridotomy (glaucoma) |  |  |  |

1. Influence of financing on the materials used for treatments

Are there any factors that significantly influence the costs that is not included in the price differentiation? (What influences changes in tariffs?)

|  | What factors differentiate the costs of the procedures? |
| --- | --- |
| Cataract |  |
| Glaucoma |  |
| Vitrectomy |  |
| Corneal transplant |  |
| AMD |  |
| DME |  |

1. The waiting time (from the first contact to receiving treatment) for services (consultations/surgeries) and the method of financing

|  | Is public funding sufficient to meet the health needs of patients requiring treatment in the following areas: (Why Y/N?) | Is there a relationship between the amount of funding and the patient's waiting time for a consultation / treatment? (Why Y/N?) |
| --- | --- | --- |
| Cataract |  |  |
| Glaucoma |  |  |
| Vitrectomy |  |  |
| Corneal transplant |  |  |
| AMD |  |  |
| DME |  |  |

1. a. Is there a centralization from the government (central govt, regional govt, health funds, other payers etc.)

b. market concentration? - are there financial incentives for concentration of services and what effect they have / do or not / why

|  | In place | Are they effective? | What could be centralized/concentrated | How to implement financial incentives? |
| --- | --- | --- | --- | --- |
| Glaucoma |  |  |  |  |
| Vitrectomy |  |  |  |  |
| Corneal transplant |  |  |  |  |
| AMD |  |  |  |  |
| DME |  |  |  |  |

1. Quality measures

|  | In place | Are they effective? | What could be measured? | What could be improved? |
| --- | --- | --- | --- | --- |
| Cataract |  |  |  |  |
| Glaucoma |  |  |  |  |
| Vitrectomy |  |  |  |  |
| Corneal transplant |  |  |  |  |
| AMD |  |  |  |  |
| DME |  |  |  |  |

1. What is your opinion on co-payments to medical procedures? Are they a barrier to services or they increase access to services? Why?

|  | Existing co-payments? | Barriers or facilitators? |
| --- | --- | --- |
| Cataract |  |  |
| Glaucoma |  |  |
| Vitrectomy |  |  |
| Corneal transplant |  |  |
| AMD |  |  |
| DME |  |  |
