# Supplementary material for: How much can we learn from each other? Polish and Hungarian good practices in financing ophthalmology care as a proposal for implementation in Ukraine
Source: PLoS One. 2024 Jul 9;19(7):e0306562. doi: 10.1371/journal.pone.0306562 (PMC11232999; doi:10.1371/journal.pone.0306562)
Supplement: S2 Table — (DOCX) [file pone.0306562.s002.docx]

**S2 Table. Interview guidebook for participants in Ukraine**

**Cataract**

| Good practice in financing mechanism that increase access for Increase number of services (availability) | Questions | Good practice in financing mechanism that increase access for Increase quality of services | Questions |
| --- | --- | --- | --- |
| Limit for funding cataract surgeries was removed. (PL, HU) | Do you think that removing restrictions on the amount of funding and financial coverage of all costs, including the costs of intraocular lenses (IOLs), for surgical treatment of cataracts is a good practice to implement in Ukraine from the point of view of expanding access to the provision of such services in the public and communal healthcare facilities sector? | Funding (higher pricing) for toric and aniridia lenses was introduced (low pricing can rule them out or result in using lower quality materials) (PL) | Is it a good practice for Ukraine to charge a higher cost of toric and aniridia lenses for patients compared to other IOLs to reduce the risk of their shortage/use of inferior quality lenses?  How should the costs of toric and aniridia lenses be covered in such a case (from the state budget or from patient co-payments)? |
| Financial incentives were introduced to shorten the hospital stay to 1-day hospitalization (PL) | Do you think that the introduction of financial incentives for medical staff/health care providers can create conditions for the introduction of "one-day" surgery and shorten the length of stay in the hospital?  Do you have experience that confirms the effectiveness of using appropriate financial incentives for the introduction of "one-day" surgery?  If such experience is available, can you share an example of incentives that were used in your health care center to introduce "one-day" surgery? | Vision impairment measurement after the surgery to assess the results of surgeries (achieved outcomes) (PL) | Do you think that as an indicator of the quality of the results of cataract surgical treatment, it is possible to measure the deterioration of vision after it? Can this indicator be used to assess the quality of the results of surgical treatment of cataracts in Ukrainian health care institutions? |
|  |  | In general, according to the interviewees and the revealed official regulation in the Hungarian compulsory health insurance scheme there is no any quality measure regarding to the ophthalmology care including cataract surgery. So, in the short future there is a highly need for the establishment such a quality indicator system, introduce before after measure test of Visual Acuity. Establish basic quality threshold in % of this Test, and plan interventions, education, input and process guality improvement programmes (HU) | Is there a need to create a system of quality indicators for the provision of ophthalmic care? |

**Glaucoma**

| Good practice in financing mechanism that increase access for Increase number of services (availability) | Questions | Good practice in financing mechanism that increase access for Increase quality of services | Questions |
| --- | --- | --- | --- |
| Large number of pharmacotherapies are reimbursed, and this ensures access to modern therapies (PL, HU) | Is it important to expand the National List of Medicines for Conservative Treatment of Glaucoma, taking into account modern treatment methods?  If so, which medicines, in your opinion, should be included in the list as a priority? | Dedicated centres for surgeries (HU) | How can establishing dedicated centres influence quality of services in the treatment of glaucoma? |

**Vitrectomy**

| Good practice in financing mechanism that increase access for Increase number of services (availability) | Questions | Good practice in financing mechanism that increase access for Increase quality of services | Questions |
| --- | --- | --- | --- |
| Several DRGs can express the cost differences between the various types of vitrectomies  (HU) | Do you think that the treatment of pathological changes in the vitreous body and retina using vitrectomy does not meet the parameter "complexity of the case - reimbursement of the supplier's costs by the NHSU" and requires changes to the relevant specifications, which requires a revision of the value of the weight coefficient of the diagnostic-related group C03 "Operations on the retina" (0.513)? | Dedicated centres for surgeries (HU) | Do you support the use of such an international practice that the treatment of pathological changes of the vitreous body and retina with the use of vitrectomy should be concentrated at the level of individual providers (for example, in cluster hospitals or specialized centers)?  If your answer is "Yes", then does this require the development of special algorithms and protocols to create an appropriate routing system for the patient? Or how do you see the organization and financing of such a model? |

**Cornea transplant**

| Good practice in financing mechanism that increase access for Increase number of services (availability) | Questions | Good practice in financing mechanism that increase access for Increase quality of services | Questions |
| --- | --- | --- | --- |
| Separate payments for transplantations and for cornea were introduced to cover the cost of procuring cornea from commercial cell & tissue banks if they are not receiving cornea from the public cell & tissue banks. This helped health providers to increase the number of transplantations by enabling them to reimburse the cost of cornea from commercial cell and tissue banks (PL)  Functioning tissue banks to have sufficient transplants (the tissue is paid to this tissue bank by hospitals from DRGs payment (HU)) | Is it appropriate to use in corneal transplantation to cover the costs of suppliers of acquiring corneas from commercial cell and tissue banks (in the experience of Poland, this model is effective for suppliers in the presence of problems in obtaining corneas from state cell and tissue banks and has contributed to the increase in the number of transplants)? | Specialized centres to carry out treatments (PL, HU) | Do you consider it a good practice to concentrate the provision of medical care for corneal transplantation at the level of specialized institutions with full coverage of costs by the state? Are there legal conditions for this? |

**AMD**

| Good practice in financing mechanism that increase access for Increase number of services (availability) | Questions | Good practice in financing mechanism that increase access for Increase quality of services | Questions |
| --- | --- | --- | --- |
| Financial products for outpatient treatment (PL) | Is it appropriate to include reimbursement for conservative treatment of age-related retinal maculopathy in the state program "Affordable Medicines"? If so, what medications can you recommend for inclusion?  Do you think that the treatment of age-related maculopathy with the use of intravitreal injections should:  a) be coded according to a separate diagnostically related group with a separate weighting factor for this group?  b) approval in the specification of the relevant requirements for carrying out such interventions? | Introduction of financial products for evaluation of achieved outcomes (PL)  Each injection is paid separately by TPP (HU, PL)  Specialized centres to carry out treatments (PL) | Do you think it is necessary to introduce a separate package in the Programme of Medical Guarantees, which will refer to the introduction of screening / prevention of this disease and singular treatments (injections)? If yes, please suggest inclusion criteria for such a package (e.g. reimbursement cost per patient, etc.)?  Do you consider treating patients with AMD in specialised centres to be a good practice? |

**DME**

| Good practice in financing mechanism that increase access for Increase number of services (availability) | Questions | Good practice in financing mechanism that increase access for Increase quality of services | Questions |
| --- | --- | --- | --- |
| Wide coverage of diagnostics for DME (HU, PL)  Financial products for outpatient treatment (PL) | Do you think that the treatment of diabetic retinopathy should:  a) to be coded according to a separate diagnostically related group with a separate weighting factor for this group depending on the stage of the disease, the complexity of the case and indications for surgical treatment (laser photocoagulation, vitrectomy)?  b) approval in the specification of relevant requirements for diagnostic and therapeutic interventions? | Specialized centres to carry out treatments (PL)  Each injection is paid separately (PL) | Do you think it is necessary to introduce a separate package in the Programme of Medical Guarantees, which will refer to the introduction of screening / prevention of this disease and singular treatments (injections)?  Do you consider treating patients with AMD in specialised centres to be a good practice? |
