# Supplementary material for: How much can we learn from each other? Polish and Hungarian good practices in financing ophthalmology care as a proposal for implementation in Ukraine
Source: PLoS One. 2024 Jul 9;19(7):e0306562. doi: 10.1371/journal.pone.0306562 (PMC11232999; doi:10.1371/journal.pone.0306562)
Supplement: S3 Table — (DOCX) [file pone.0306562.s003.docx]

**S3 Table. Financial Mechanisms for ophthalmology treatment (2019)**

| Country | Poland | Hungary | Ukraine |
| --- | --- | --- | --- |
| Cataract surgery | 2 DRGs (B18- “complicated” cases and children, B19-other), 426 euro-500 euro  Additional indicators in case of toric or aniridia lens  Decrease in payment (-10%) if a share of one-day cases less than 80%  No financial limits, financed on a fee-for-service basis | 4 DRGs (flexible artificial lens, toric lens, bilateral simultaneous surgery, without reimbursement of the price of the artificial lens),  414 euro – 494 euro (318 euro without lens)  It is reimbursed for a certain annual volume limit set by the NHIF at the level of healthcare institutions.  No financial limits in fee-for-service based on private or public service providers. | Hospital care in ophthalmology is provided in hospitals with budgets for all services and individual pricing for services.  While publicly financed health services are officially free at  the point of service, there are gaps in public coverage that  result in large costs to patients |
| Glaucoma surgery | 4 DRGs (B11 - Comprehensive cataract and glaucoma treatments; B72 - Major procedures in glaucoma and choroid; B73 - Moderate procedures in glaucoma and choroid; B74 - Small procedures in glaucoma and choroid):  42 euro-785 euro  Additional price indicator (+10%) in case of more than 250 complicated surgeries (B11, B72)  Payment up to the hospital budget (for hospital network entities) or the ophthalmology clinic budget (for other entities) | There is no special DRG for Glaucoma care: 02 074C – Eye other diseases  Normative day: 5 days, (minimum 3 days)  Weight number: 0.3873 (Tariff by NHIF: 76,7 th HUF = 220 EUR)  Minimally invasive principle: to avoid surgery, and to treat the patients in an ambulatory care setting (out-patient clinics, hospitals, University clinics). Responsibility of the public sector to give complex long-term solutions via regular specialist control and effective eye drop therapy.  It is reimbursed for a certain annual volume limit set by the NHIF at the level of healthcare institutions. |  |
| Vitrectomy | 4 DRGs (B16 - procedures with a vitrectomy using silicone oil or decalin, including multi-procedure, B16G - procedures with phakovitrectomy using silicone oil or decalin, including multi-procedure, B17 - procedures with vitrectomy, including multi-procedure surgeries, B17G - procedures with phakovitrectomy, including multi-procedure); 1464-1894 euro  Additional price indicator (+10%) in case of more than 400 surgeries  Payment up to the hospital budget (for hospital network entities) or the ophthalmology clinic budget (for other entities) | 3 DRGs:  - 069A-Pars plana vitrectomy with artificial lenses (RW: 3.05, appr. 1600 EUR)  - 069B-Pars plana vitrectomy (RW: 2.73, appr. 1450 EUR  - 069C-Other vitrectomies (RW: 1.05, appr. 560 EUR).  There are no financial or volume limits. The directors are responsible, or head of a department for how many human resources and technical efforts can dedicate to these interventions within the ophthalmic unit. |  |
| AMD | Payment for each visit, which consists of separate financial products:  Module 1: (1) visit (and medical tests for putting into drug programme only once) 80 euro + (2) injection 70 euro + (3) drug costs  or Module 2: (1) visit for evaluation of achieved outcomes 40 euro  Payment up the budget defined for the AMD drug programme | DRG 0633-Aged related macula degeneration treatment with neuro-vascularization (RW: 0.15, appr. 80 EUR)  In a one-day surgery setting:  12219 Photodynamic treatment of elderly macular degeneration with neuro-vascularization.  12220 Treatment of elderly macular degeneration with neovascularization by intravitreal injection  It is reimbursed for a certain annual volume limit set by the NHIF at the level of healthcare institutions. |  |
| DME | Payment for each visit, which consists of separate financial products:  Module 1: (1) visit (and medical tests for putting into drug programme only once) 80 euro + (2) injection 70 euro + (3) drug costs  or Module 2: (1) visit for evaluation of achieved outcomes 40 euro  Payment up to the budget defined for the DME drug programme | There is no special DRG for DME:  074C – Eye other diseases.  Normative day: 5 days, (minimum 3 days)  RW: 0,3873 (Tariff by NHIF: 76,7 th HUF = 220 EUR)  It is reimbursed for a certain annual volume limit set by the NHIF at the level of healthcare institutions. |  |
| Cornea transplantations | 3 DRGs (B04-Category I, B05-Category II, B06-Category III); 918 - 1699 euro  Payment up to the hospital budget (for hospital network entities) or the ophthalmology clinic budget (for other entities) | There are special DRGs:  0610-Cornea transplant (RW: 1.6, appr. 850 EUR)  0611-Cornea transplant with artificial lens implantation (RW: 1.85, appr. 1000 EUR.)  There are no financial or volume limits. The number of procedures depends on the number of donor lenses. |  |

Source: own work.
