# Supplementary material for: How much can we learn from each other? Polish and Hungarian good practices in financing ophthalmology care as a proposal for implementation in Ukraine
Source: PLoS One. 2024 Jul 9;19(7):e0306562. doi: 10.1371/journal.pone.0306562 (PMC11232999; doi:10.1371/journal.pone.0306562)
Supplement: S4 Table — (DOCX) [file pone.0306562.s004.docx]

**S4 Table**. **Cataract volumes (% change to the previous year) (in thousands)**

|  | 2016 | 2017 | 2018 | 2019 | 2020 | 2021 |
| --- | --- | --- | --- | --- | --- | --- |
| Poland | 245 | 300  (+22%) | 314  (+5%) | 355  (+13%) | 234  (-34%) |  |
| Hungary | 58,3 | 60,0  (+3,1%) | 64,5  (+7,4%) | 66,6  (+3,3%) | 42,9  (-64,4%) | 32,6  (76,5%) |

Source: own work based on National Health Insurance databases (www.nfz.gov.pl; www.neak.gov.hu)
